# Supplementary material for: Coral restoration is only practical when local human pressure levels are low
Source: PLoS One. 2026 Sep 2;21(9):e0355728. doi: 10.1371/journal.pone.0355728 (PMC13537597; doi:10.1371/journal.pone.0355728)
Supplement: Supplementary file 1 [file pone.0355728.s001.pdf]

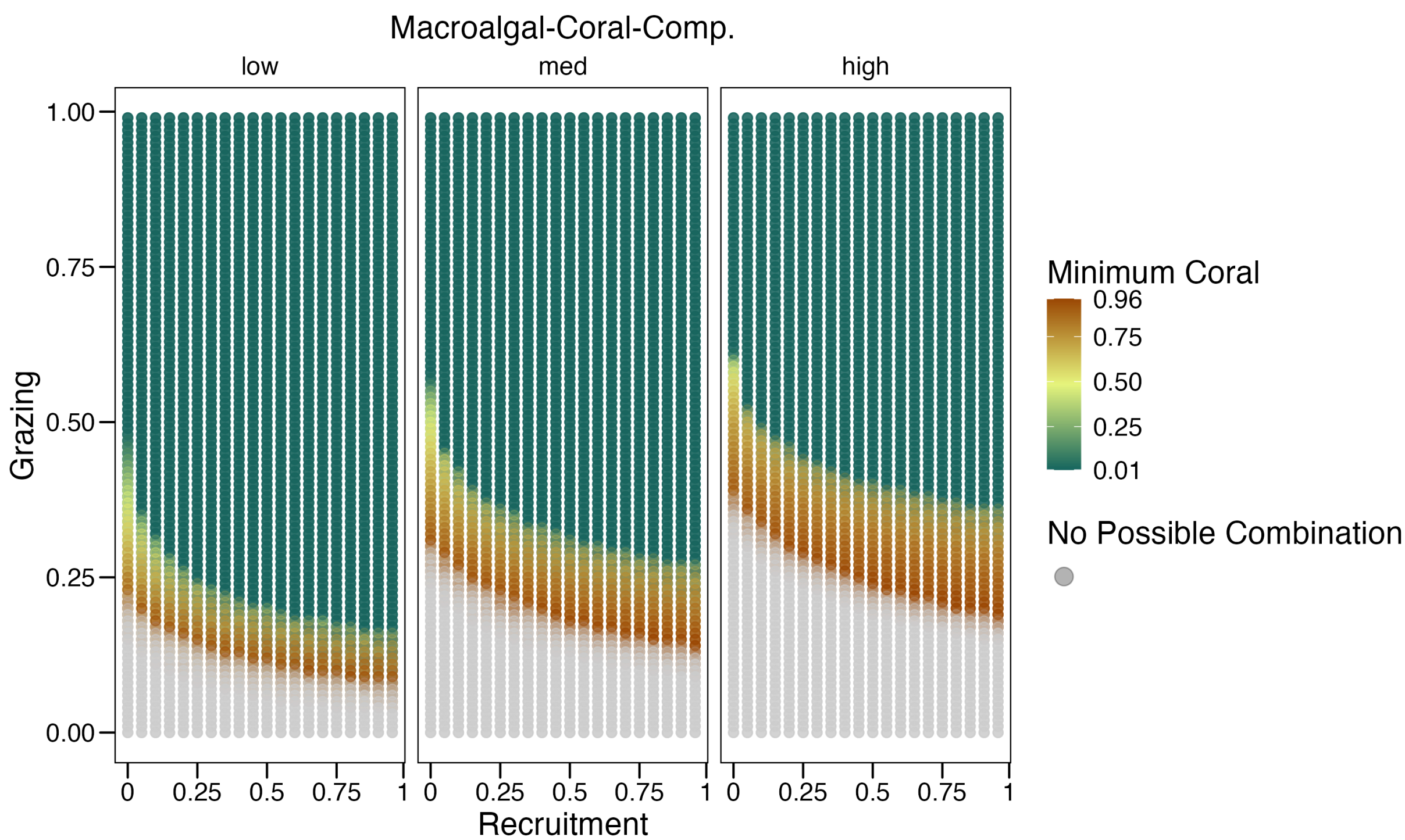

Figure S1: Minimum amount of coral cover (percentage) required, given a particular parameter combination, for a given trajectory to result in the reef being in a "healthy self-sustaining coral reef state" (i.e., >30% coral cover & <30% macroalgae cover). Light grey points indicate that no possible parameter combination or initial condition will result in the reef being in a "healthy self-sustaining coral reef state".
