# Supplementary material for: Coral restoration is only practical when local human pressure levels are low
Source: PLoS One. 2026 Sep 2;21(9):e0355728. doi: 10.1371/journal.pone.0355728 (PMC13537597; doi:10.1371/journal.pone.0355728)
Supplement: Supplementary file 4 [file pone.0355728.s004.pdf]

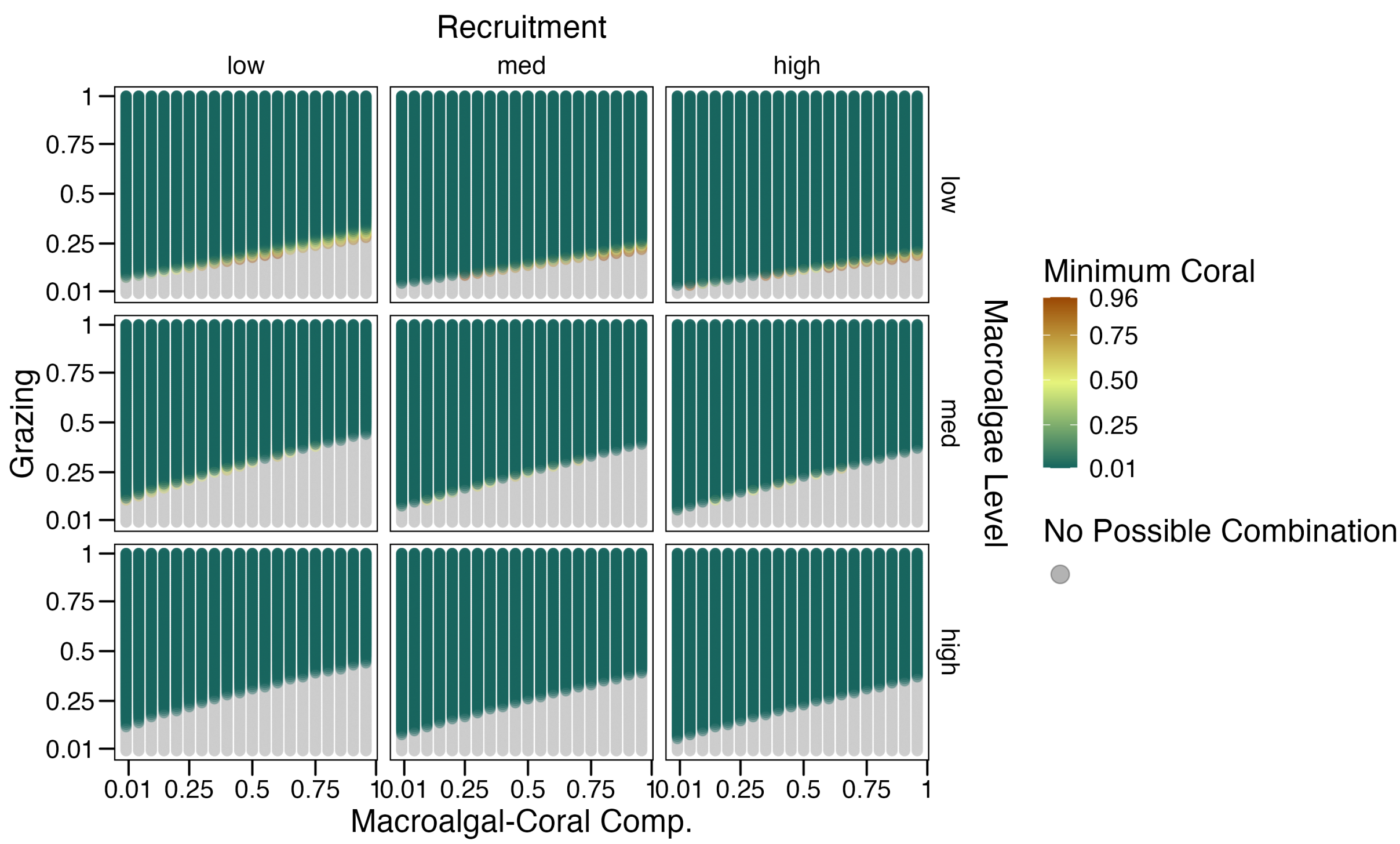

Figure S4: Each point represents the minimum amount of initial coral cover for the given parameter combinations, such that certain initial values of macroalgae result in the reef being in a "healthy self-sustaining coral reef state" (i.e., trending towards a stable state with >30% coral cover & <30% macroalgae cover). The values on the right y-axis depict whether the initial values of macroalgae considered are low (0-33%), medium (33-66%) or high (>66%). Light grey points indicate that no possible parameter combination or initial condition results in the reef being in a "healthy self-sustaining coral reef state".
